# The Effects of Four Different Thawing Methods on Quality Indicators of *Amphioctopus neglectus*

**DOI:** 10.3390/foods13081234

**Published:** 2024-04-17

**Authors:** Huixin Zhang, Shuji Liu, Shuigen Li, Xiaoe Chen, Min Xu, Yongchang Su, Kun Qiao, Xiaoting Chen, Bei Chen, Hong Zhong, Hetong Lin, Zhiyu Liu

**Affiliations:** 1 College of Food Science, Fujian Agriculture and Forestry University, Fuzhou 350002, China; 15080501621@163.com (H.Z.); hetonglin@163.com (H.L.); 2 Fisheries Research Institute of Fujian, Xiamen 361013, China; xumin@jmu.edu.cn (M.X.); suyongchang@stu.hqu.edu.cn (Y.S.); qiaokun@xmu.edu.cn (K.Q.); 13950001893@163.com (B.C.); 3 Fujian Fisheries Technical Extension Station, Fuzhou 350002, China; 2201020770@fjmu.edu.cn; 4 College of Food and Pharmacy, Zhejiang Ocean University, State Key Laboratory of Aquatic Products Processing of Zhejiang Province, Zhoushan 316022, China; xiaoechen@163.com; 5 Key Laboratory of Cultivation and High-Value Utilization of Marine Organisms in Fujian Province, National Research and Development Center for Marine Fish Processing (Xiamen), Xiamen 361013, China; xtchen@jmu.edu.cn; 6 Dongshan Paul Food Co., Ltd., Zhangzhou 363400, China; zsfoctopus@163.com

**Keywords:** octopus, thawing methods, quality indicators, water retention, protein oxidation

## Abstract

*Amphioctopus neglectus* is a species of octopus that is favored by consumers due to its rich nutrient profile. To investigate the influence of different thawing methods on the quality of octopus meat, we employed four distinct thawing methods: air thawing (AT), hydrostatic thawing (HT), flowing water thawing (FWT), and microwave thawing (MT). We then explored the differences in texture, color, water retention, pH, total volatile basic nitrogen (TVB–N), total sulfhydryl content, Ca^2+^–ATPase activity, and myofibrillar protein, among other quality indicators in response to these methods, and used a low-field nuclear magnetic resonance analyzer to assess the water migration that occurred during the thawing process. The results revealed that AT had the longest thawing time, leading to oxidation-induced protein denaturation, myofibrillar protein damage, and a significant decrease in water retention. Additionally, when this method was utilized, the content of TVB–N was significantly higher than in the other three groups. HT, to a certain extent, isolated the oxygen in the meat and thus alleviated protein oxidation, allowing higher levels of Ca^2+^–ATPase activity, sulfhydryl content, and springiness to be maintained. However, HT had a longer duration: 2.95 times that of FWT, resulting in a 9.84% higher cooking loss and a 28.21% higher TVB–N content compared to FWT. MT had the shortest thawing time, yielding the lowest content of TVB–N. However, uneven heating and in some cases overcooking occurred, severely damaging the protein structure, with a concurrent increase in thawing loss, W value, hardness, and shear force. Meanwhile, FWT improved the L^*^, W^*^ and b^*^ values of octopus meat, enhancing its color and water retention. The myofibrillar protein (MP) concentration was also the highest after FWT, with clearer subunit bands in SDS-PAGE electrophoresis, indicating that less degradation occurred and allowing greater springiness, increased Ca^2+^–ATPase activity, and a higher sulfhydryl content to be maintained. This suggests that FWT has an inhibitory effect on oxidation, alleviating protein oxidation degradation and preserving the quality of the meat. In conclusion, FWT outperformed the other three thawing methods, effectively minimizing adverse changes during thawing and successfully maintaining the quality of octopus meat.

## 1. Introduction

*Amphioctopus neglectus* is a type of octopus that belongs to the class Cephalopoda, order Octopoda, and family Octopodidae. It can be found in a range of locations in the Bohai Sea, Yellow Sea, East China Sea, and South China Sea, from the surface to the deep sea, and from the intertidal zone and continental shelf to the continental slope [1].

Octopus meat is rich in proteins, minerals, amino acids, and unsaturated fatty acids, especially DHA and EPA [2]. Studies have shown that octopus polypeptides, phospholipids, and octopamine extracted from octopi have the effects of lowering blood lipids, reducing blood pressure, and preventing cardiovascular diseases, in addition to its antioxidant and anti-inflammatory effects [3]. In one study, Chakraborty et al. [4] conducted bioassay-guided fractionation of an ethyl acetate/methanol extract obtained from *Amphioctopus neglectus*, demonstrating its free radical-scavenging potential and antihypertensive abilities. At present, octopus meat is primarily sold fresh or frozen, with a small amount being processed into canned or dried products; however, its short preservation time when fresh limits its sales opportunities, which means that in most cases, octopi are frozen immediately after capture. Regrettably, ice crystals formed during freezing frequently trigger protein denaturation, cell membrane rupture, and temperature fluctuations, leading to recrystallization. These factors collectively diminish the product’s quality [5]. It is crucial to identify suitable thawing methods to avoid this type of damage. However, research in this area is currently limited.

Thawing loss is influenced by various factors, including the time, temperature, and method [6] of thawing. Throughout the thawing process, variations in temperature, microbial growth, and heightened enzyme activity may influence the size and distribution of ice crystals both within and outside cells, alongside the denaturation of myofibrillar proteins, ultimately resulting in a decline in quality [7,8]. Common thawing methods include air, hydrostatic, running water, and microwave thawing, all of which have different effects on sample quality. Diao et al. [9] studied the impact of low-temperature, hydrostatic water, ultrasonic, and air thawing on the quality of Antarctic krill meat, and found that hydrostatic water thawing at 15 °C better maintained the quality of the meat. In another study, Zhou et al. [10] analyzed the quality of mackerel thawed in five different ways and discovered that while microwave thawing was the fastest method, the heating was uneven, and some of the meat matured as a result. Running water thawing provided the best water retention and was found to be more suitable for thawing frozen mackerel. Ying et al. [11] researched six methods of thawing cuttlefish and concluded that saline solution and ultrasound water thawing were the most suitable, with samples thawed in saline solution demonstrating superior hardness and chewiness. In general, consumers prefer seafood with minimal juice loss and a firm texture. During thawing, water-holding capacity and texture characteristics such as hardness, chewiness, and elasticity undergo varying degrees of change [8] and interaction, affecting the taste of seafood [9]. Moreover, protein denaturation can alter the water-holding capacity and texture characteristics of the product [12]. Currently, there is limited research on the different ways of thawing octopus meat and the findings are inconsistent. Therefore, it is necessary to research the influence of thawing methods on the water-holding capacity and texture characteristics of frozen *A. neglectus* to provide a theoretical basis for selecting the optimal thawing method.

In this study, we employed air thawing (AT), hydrostatic thawing (HT), flowing-water thawing (FWT), and microwave thawing (MT) to process frozen *A. neglectus*. The effects of the different methods on the texture, water-holding capacity, and protein oxidation of octopi were investigated to identify the optimal thawing methods.

## 2. Materials and Methods

### 2.1. Materials and Reagents

Frozen *A. neglectus*, weighing (20 ± 5) g each were purchased from Dongshan Boguang Tianxing Food Co., Ltd., in Zhangzhou City, Fujian Province, China. After removal of the internal organs in the processing factory, they were placed in low-temperature conditions, transported to the laboratory, then stored at −20 °C until use.

We also purchased a SDS-PAGE gel rapid preparation kit (12.5%) from Affinity (Wuhan, China) Life Science & Technology Co., Ltd.; a Micro total sulfhydryl group kit and a micro Ca^2+^–ATPase kit from Beijing Solaibao Technology Co., Ltd., Beijing, China; a Bradford method protein detection kit from Biyuntian Biotechnology Co., Ltd., Shanghai, China; and bromophenol blue, methyl red (analytical grade), anhydrous ethanol (analytical grade), sodium chloride (analytical grade), and sodium hydroxide (analytical grade) from Sinopsin Group Chemical Reagent Co., Ltd, Shanghai, China.

### 2.2. Methods

#### 2.2.1. Sample Pretreatment

The frozen octopi were removed and randomly divided into four groups, then underwent wither air thawing (AT), hydrostatic thawing (HT), flowing-water thawing (FWT), or microwave thawing (MT). The specific thawing procedures are outlined in Table 1. During the thawing process, a thermocouple probe (BD-TC-SC, Shenzhen, China) was inserted into the center of each octopus. The thawing was considered to be complete when the central temperature reached 4 °C [13]. Various indicators were measured at this stage of the process.

#### 2.2.2. Thawing Loss Rate

The mass of each octopus was measured prior to thawing (*W*_1_/g). After the thawing was complete, absorbent paper was used to remove surface moisture from the octopus, then the mass was measured a second time (*W*_2_/g). The thawing loss rate (*Tlr*) was calculated according to Formula (1).

(1) $$Tlr/\%=\frac{W_{1}-W_{2}}{W_{1}}\times 100$$

#### 2.2.3. Water-Holding Capacity

We adhered to the method proposed by Jiang et al. [14], with a slight modification. After the thawing process was complete, the dried octopus was weighed (*W*_3_/g), wrapped in filter paper, and placed in a 50 mL centrifuge tube, followed by 20 min centrifugation at 4 °C and 10,000 r/min (Eppendorf 5810R, Hamburg, Germany). After that, the sample was removed, the filter paper was stripped away, and the mass of the octopus was reweighed (*W*_4_/g). The water-holding capacity (*Whc*) was calculate according to Formula (2).

(2) $$Whc/\%=\frac{W_{3}-W_{4}}{W_{4}}\times 100$$

#### 2.2.4. Cooking Loss Rate

As described by Wang et al. [15], but with minor adjustments, we weighed the thawed octopus (*W*_5_/g), heated it in a water bath at 90 °C for 30 min, then cooled it to room temperature. The surface moisture was removed with absorbent paper. Then, the mass was weighed again (*W*_6_/g), and the cooking loss rate (*Clr*) was determined according to Formula (3).

(3) $$Clr/\%=\frac{W_{5}-W_{6}}{W_{5}}\times 100$$

#### 2.2.5. pH

To test the pH, we weighed out 5 g of thawed octopus, placed it in a 100 mL beaker, added 45 mL of distilled water, homogenized it for 30 s in a homogenizer, let it stand for 30 min, then filtered the resulting solution. After that, we measured the pH value of the filtrate (FE28 pH, Zurich, Switzerland).

#### 2.2.6. Color

Implementing the method of Wen et al. [16] with slight modifications, we used an automatic colorimeter (BGZ-240 ADCI, Beijing, China) to measure the central 1.5 cm × 1.5 cm area of the head of the octopus, then measured the value of L^*^ (brightness), a^*^ (red-green chromaticity), and b^*^ (yellow-blue chromaticity). A white board was used to correct the colorimeter before each measurement. Then, we calculated the whiteness value (W) according to Formula (4).

(4) $$W=100-\sqrt{{\left(100-L^{*}\right)}^{2}+{\left(a^{*}\right)}^{2}{+(b^{*})}^{2}}$$

#### 2.2.7. Texture

Referencing the work of Torres et al. [17], we dried the thawed octopus with absorbent paper and measured the width of its meat (2.5 cm × 1.0 cm × 0.5 cm) using an A/MORS P/5S probe of texture analyzer (TA–XTplus, Stable Micro System, Surrey, UK) with a pre-test speed of 3 mm/s, a test speed of 1 mm/s, a post-test speed of 1 mm/s, a compression ratio of 50%, and a 5-second interval between two compressions. Each group of samples was measured six times.

#### 2.2.8. Volatile Basic Nitrogen (TVB–N) Value

The thawed octopus was crushed, and a 20 g portion was soaked in 100 g water for 30 min and filtered for use. The detection method used was the micro-diffusion method described in GB 5009.228-2016 National Food Safety Standard Determination of Volatile Basic Nitrogen in Food [18].

#### 2.2.9. Extraction and Determination of Mass Concentration of Myofibrillar Protein

The extraction method for myofibrillar proteins followed the procedure described by Benjakul et al. [19], with slight modifications. The thawed octopus was crushed and homogenized with four times the volume of 10 mmol/L Tris–HCl (pH 7.2). After 20 s of homogenization, the mixture was centrifuged at 4500 rpm for 20 min. The precipitate was then re-suspended in four times the volume of 10 mmol Tris–HCl buffer (containing 0.6 mol/L NaCl, pH 7.2), homogenized for 90 s, and centrifuged at 4500 rpm for 20 min. The resulting supernatant, was considered to be the myofibrillar protein solution.

The extracted myofibrillar protein solution was diluted to 1.00 mg/mL, mixed with an equal volume of 2 × SDS–PAGE buffer, boiled in a water bath for 3~5 min, and then centrifuged at 10,000 rpm for 1 min. The supernatant was used as the electrophoresis sample. A 12% separating gel and a 5% stacking gel were prepared. Electrophoresis was conducted at a voltage of 200 V until the tracking dye, Coomassie Brilliant Blue R 250, reached the bottom of the gel. Staining took place for 5~6 h, after which the gel was photographed and analyzed using a scanner (Epson perfection V 700, Nagano Prefecture, Japan). The concentration of myofibrillar proteins was determined using the Bradford protein assay kit.

#### 2.2.10. Determination the Activity of Ca^2+^–ATPase

The Ca^2+^–ATPase activity of thawed octopi was measured using the microscale Ca^2+^–ATPase activity assay kit.

#### 2.2.11. Determination of Total Sulfhydryl Content

The total sulfhydryl content of thawed octopi was quantified using the microsulfhydryl assay kit, and the results were expressed in terms of protein mass.

#### 2.2.12. Water Distribution

Following the method of Ge et al. [20], we analyzed and determined the distribution of water content using low-field nuclear magnetic resonance (LF–NMR) technology (MesoMR 23-060H-I, Suzhou, China). The thawed octopus meat was cut into 2.5 cm × 1.0 cm × 0.5 cm samples, after which we utilized a 40 mm nuclear magnetic detection tube with the following parameters: SW = 200 kHz, RG1 = 20, P1 = 18.00 μs, DRG1 = 3, TD = 160,014, PRG = 1, TW = 2000 ms, NS = 8, P2 = 37 μs, TE = 0.400, NECH = 4000. After that, we generated transverse relaxation time (*T*_2_) spectra through iterative inversion.

#### 2.2.13. Data Processing and Analysis

Unless otherwise specified, the experiments were conducted in triplicate and the results were presented as mean values ± standard deviation. All data were subjected to SPSS 25.0 by one-way ANOVA and post hoc testing for comparison of the differences between means, and the differences were considered significant at 5% (*p* < 0.05). The graphical representation was created using Origin 2018 software.

## 3. Results

### 3.1. Effects of Thawing Methods on the Thawing Time and Water Retention of A. neglectus

As shown in Table 2, microwave thawing (MT) was the fastest method, which took 1/20 of the time of air thawing (AT), and 1/2 of the time of flowing-water thawing (FWT). MT was the fastest due to the intense oscillation and friction of polar groups in frozen octopi under the alternating electric field of microwaves, converting microwave energy into heat [21]. With strong penetration ability at low temperatures, MT required the shortest time: only (20 ± 1.63) min, which was significantly less than that taken by AT and HT (*p* < 0.05).

The second most effective method was flowing-water thawing (FWT), which had a higher heat capacity than air thawing, resulting in faster heat transfer and a significant reduction in thawing time [22]. Hydrostatic thawing (HT), which has a higher heat capacity, also accelerated the heat exchange and reduced the thawing time. Although microwave thawing (MT) was the quickest, the octopus meat was often scorched during this process due to the uneven thickness and shape of the octopus [23].

During thawing, the ice crystals frozen inside the muscles melt into water, and some are reabsorbed by the muscle fibers. If mechanical damage to the muscle occurs during the thawing process, it may lead to leakage of water-soluble nutrients such as amino acids and vitamins, with a concomitant decrease in product quality and commercial value [24]. Both the thawing loss rate and the cooking loss rate significantly influence the water retention capacity of frozen aquatic products [25,26]. As shown in Table 2, the thawing loss rate and cooking loss rate were highest in the MT group, which were 22.03% and 6.25% higher than those in the FWT group, respectively. This was consistent with the results of Tan Mingtang et al. [27]. The main reason for this is that the microwave caused uneven distribution of the polar water molecules in the muscle, leading to disparate absorption of heat energy in different parts of the octopus. This resulted in uneven thawing and severe damage to the protein structure, preventing melted water from binding with proteins [28]. On the other hand, flowing-water thawing (FWT) had the lowest thawing loss rate and cooking loss rate but the highest water retention capacity. This may be attributed to the fact that FWT, compared to HT, passed through the ice crystal melting zone more quickly (−5~0 °C) and decreased microbial reproduction. This partially alleviated the degradation of muscle proteins and maintained the water retention capacity of the muscles [29].

The impact of the four thawing methods on the water retention capacity of octopus muscles was ranked as follows: FWT > MT > HT > AT, with FWT having the highest water retention capacity—9.33% higher than that of AT. The influence on the cooking loss rate followed the order of AT > HT > MT > FWT, in which AT was 16.15% higher than FWT, contradicting the trend of water retention capacity. The findings were similar to the results of Lv et al. [11], which may be attributed to the long duration of air thawing. Again, this may be attributed to the extra time taken for defrosting to occur in open air, providing a breeding ground for microorganisms. As a result, during this process, proteins break down and do not hydrate well with reosmotic water [30]. Uneven microwave heating destroyed the muscle fiber structure, significantly affecting both the water retention and cooking loss rates (*p* < 0.05).

In summary, FWT was proved to demonstrate superior water retention. The swift transition through the ice crystal melting zone in FWT minimizes microbial growth and reduces the time available for protein degradation, thus protecting the muscles’ water-binding capacity [22]. In addition, the economic cost of the four thawing methods was calculated. AT took the longest time; HT required a certain amount of time and water; FWT consumed the most water, but the time was relatively short; and MT had the shortest time but the highest power consumption. Overall, the economic cost of the four thawing methods was not very different.

### 3.2. Effects of Thawing Mode on Water Distribution of A. neglectus

**Figure 1:**
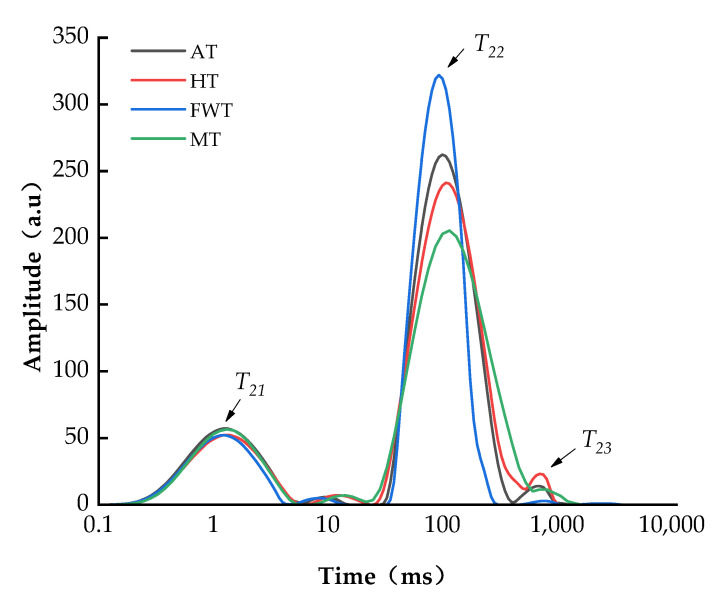
Effects of different thawing methods on the *T*_2_ relaxation time of *A. neglectus*.

**Figure 2:**
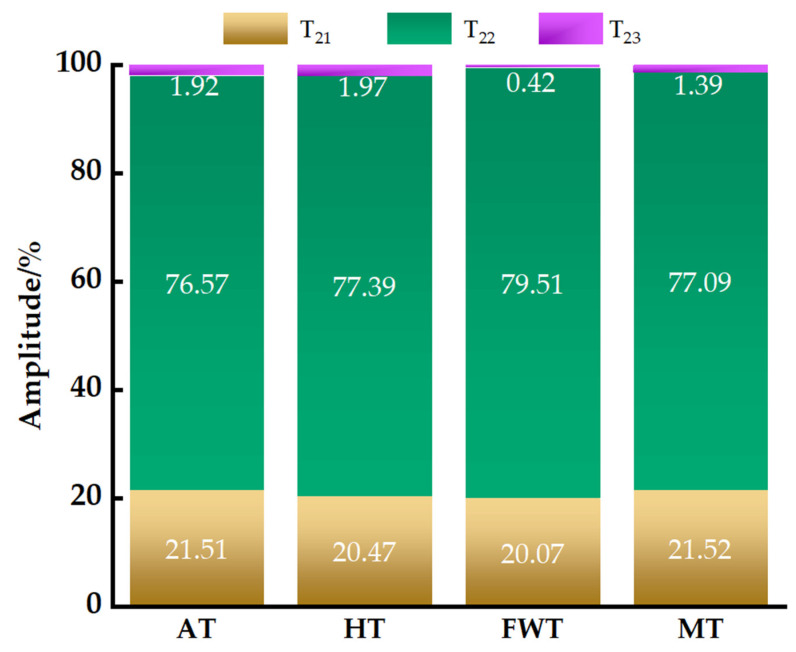
The variation in moisture content in different states of *A. neglectus* after the application of different thawing methods.

The transverse relaxation time (*T*_2_) of LF–NMR reflects the distribution of water in the sample. The longer the relaxation time, the looser the combination of water with substrates, indicating greater water mobility [31]. In octopi, water can be categorized into bound water, which is contained within tightly bound protein macromolecules (*T*_21_, 0~10 ms), immobilized water, which is located between muscle fibers and membranes (*T*_22_, 10~100 ms), and free water, which is found outside muscle fibers or extracellularly (*T*_23_, 100~1000 ms) [32]. Of these, *T*_23_ has the longest relaxation time, and is involved in enzymatic reactions and biochemical processes in food [20]. The oxidation of proteins exposes internal hydrophobic groups, reducing their capacity to bind with water and ultimately increasing both their mobility and ease of removal. Meanwhile, a lower degree of protein oxidation results in a tighter binding of water molecules [19]. Relaxation time graphs for these three water forms observed after the application of different thawing methods are shown in Figure 1 and Figure 2, demonstrating that octopus muscle thawed with all four methods contained three types of water: bound, immobilized, and free water. Of these, immobilized water constituted the majority of total water forms and was the primary water component in octopus muscle, consistent with the findings of Li et al. [33]. The FWT group showed the highest peak for *T*_22_, indicating the highest content of immobilized water, and thus, superior water retention. This finding aligned with the results obtained for water-holding capacity and cooking loss. Also, the content of *T*_23_ was determined the lowest in the FWT group, indicating that FWT had the best water retention. The content of *T*_23_ in AT and HT was more than in MT, possibly due to the longer thawing time. Increased protein oxidation took place, causing more severe impairment to the myofibrillar protein matrix, and eventually leading to the conversion of immobilized water molecules into their unbound, free-flowing form [29]. The relative T_21_ content was not significant for any of the four thawing methods (*p* > 0.05), indicating that the bound water remained in a relatively stable state with little variation. This may be because bound water consists of water molecules tightly bound to polar groups inside the octopus cells through covalent bonds, making it less prone to loss [27]. The T_22_ content of AT differed by 2.94% from that of FWT, suggesting that, under AT treatment, both the water-binding capability and water retention of octopi was poorer. This may be attributed to the prolonged thawing time in the AT group, and the resulting damage to the muscle fiber proteins [20].

In summary, the water state of octopi in the FWT group was relatively stable, exhibiting excellent water retention. This finding was consistent with the results of water retention capacity and cooking loss, and similar results were reported by Tan et al. [27].

### 3.3. Impact of Thawing Methods on the Color and pH of A. neglectus

Color is the external manifestation of physiological, biochemical, and microbial changes in muscles, influencing the customer acceptance level and serving as a subjective indicator of food spoilage [30]. As shown in Table 3, among the four different thawing methods, the brightness value (L^*^) of octopi in both the FWT and HT groups was relatively high, with FWT having the highest L^*^ value, 1.28 times that of the MT group. In contrast, MT had the lowest L^*^ value, indicating poor gloss. This may be attributed to uneven microwave heating and high temperatures, which resulted in partial muscle coagulation and a decrease in gloss [10]. The redness value a^*^ of AT was significantly lower than that of the other three groups, mainly because of the long time taken to achieve AT leading to severe oxidation of oxygenated myoglobin and a significant reduction in the a^*^ value. Jiang et al. [34] found that carbonyl compounds formed by protein oxidation easily react with amino acid compounds to produce dark-colored substances. The MT and HT groups had the highest a^*^ value, which may be caused by the protein denaturation caused by high temperatures during microwave thawing and prolonged exposure to air during air thawing. The rapid increase in temperature during MT resulted in serious protein oxidation, significantly reducing the b^*^ value to the lowest of all the thawing methods. Possibly due to the prolonged thawing time and the oxidation of protein, the b^*^ value of AT was significantly lower than HT and FWT (*p* < 0.05).

The whiteness value (W) of food is related to thawing loss and internal oxidation degree. The thawing loss rate was the highest for MT, as a large amount of water was lost during this process, and consequently, the W value was significantly reduced. This was consistent with the results of Xia et al. [35], indicating that MT had the most damaging effect on the quality of octopi [11]. The W value after FWT was higher than that of the AT, HT, and MT groups, possibly due to the lower protein oxidation.

During the initial stage of thawing, muscle glycogen may break down and oxidate, leading to the accumulation of lactic acid and inorganic phosphoric acid, and the degradation of adenosine triphosphate (ATP), resulting in a decrease in pH value [36]. With a prolonged thawing time, proteins decompose into alkaline substances, with a resulting gradual increase in the pH value [6]. As shown in Table 3, the long period of time taken to achieve AT (400 min) resulted in proteins decomposing into alkaline substances, demonstrating a significantly higher pH value compared to the other groups (*p* < 0.05), followed by HT and MT, and the pH value in FWT was the lowest. For HT, this may be attributed to the extended thawing time (115 min), whereas for MT, the high temperature accelerated the degradation of proteins, and therefore the production of peptides and amines. In short, FWT-treated octopi exhibited optimal color and pH levels, indicating better freshness and less oxidation.

### 3.4. Effects of Thawing Methods on the Texture of A. neglectus

Texture characteristics are a series of parameters that describe and reflect the sensory qualities and consistency of food. Physical tests, sensory evaluations, or instrumental analyses can be employed to measure attributes such as chewiness, elasticity, cohesiveness, hardness, shear force, etc. Hardness refers to the energy exerted during the chewing process, while shear force reflects the chemical structural status of muscle proteins during the thawing process [37].

**Figure 3:**
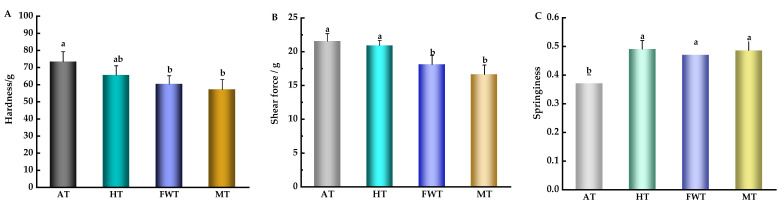
Effects of different thawing methods on the hardness (**A**), shear force (**B**), and springiness (**C**) of *A. neglectus* (^a,b^ indicate that different thawing treatments have significant differences, *p* < 0.05).

Figure 3A shows that the hardness of octopus muscle was the highest in the AT group, which was significantly higher than that of the FWT and MT groups (*p* < 0.05), but showed no significant difference from the HT group (*p* > 0.05). This result aligned with the findings of Tan et al. [27], and may be attributed to the fact that the FWT and MT methods caused a higher degree of denaturation in the muscle fiber proteins, leading to reduced intercellular binding forces and a softer muscle texture. Similarly, the shear force of octopus muscle varied among the four thawing methods (Figure 3B). The shear force of octopus muscle in the FWT and MT groups was significantly lower than in the AT and HT groups, indicating that the FWT and MT methods resulted in a softer muscle texture. This was due to the greater hardness of octopus muscle in the AT and HT groups, which required a higher shear force. Another parameter, springiness, indicates the degree to which fish meat regains its shape after external force is removed; the greater the springiness value, the better the texture [16]. In Figure 3C, the springiness of the AT group was the lowest at only 0.37, which is significantly lower than the other three groups (*p* < 0.05), indicating that the texture of MT was compromised due to increased hardness and shear force. Therefore, the texture analysis revealed that FWT resulted in a more tender product with lower hardness and shear force, suggesting a more desirable mouthfeel.

### 3.5. Effects of Thawing Mode on the Content of Total Volatile Basic Nitrogen (TVB–N) in A. neglectus Muscle

**Figure 4:**
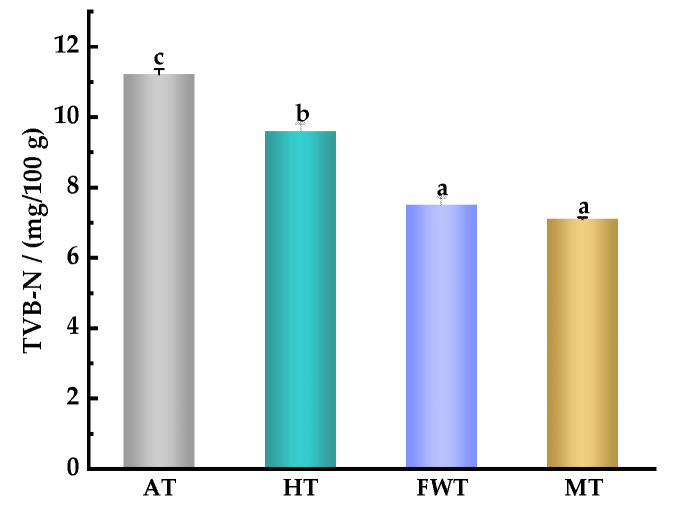
Effects of different thawing methods on the content of TVB–N in *A. neglectus* (^a,b,c^ indicate that different thawing treatments have significant differences, *p* < 0.05).

Total volatile base nitrogen (TVB–N) refers to the alkaline substances, including ammonia and amines, produced as a result of the decomposition of proteins in meat due to microbial or enzymatic activity during processing and storage. The TVB–N content is a common indicator used to assess the freshness of meat. A higher TVB–N value indicates a greater degree of protein degradation and, consequently, poorer freshness [38]. Microbial metabolism and the activity of endogenous enzymes have a significant impact on TVB–N values. According to the national standard GB 2733-2015 Fresh and Frozen Animal Aquatic Products [39], a TVB–N value ≤ 30 mg/100 g is considered acceptable. As shown in Figure 4, The TVB–N value in the AT group was significantly higher than that in the other three groups (*p* < 0.05), and was 1.17, 1.50, and 1.58 times that in the HT, FWT, and MT groups, respectively This was consistent with the experimental results of Li et al. [40], who investigated the effects of four thawing methods (4 °C, 3.8 kV high-voltage electrostatic field thawing, HT, FWT, and AT) on the mantle muscle of Japanese flying squid. This may be because the prolonged exposure of meat to air during the thawing process leads to severe protein damage [13]. In contrast, the TVB–N values in the FWT and MT groups were lower, with no significant difference between them (*p* > 0.05), possibly due to differences in thawing efficiency, with MT having the shortest thawing time. Consequently, there was a reduction in protein degradation caused by endogenous enzymes, and fewer microbial activities were observed, resulting in decreased production of nitrogen-containing alkaline substances like ammonia and amines [38]. FWT, which had a shorter thawing time and less microbial activity, also resulted in less TVB–N production. In summary, both AT and HT resulted in higher TVB–N values, indicating less freshness. However, FWT and MT can effectively delay the production of nitrogen-containing alkaline substances such as ammonia and amines in octopus muscle, better preserving its freshness.

### 3.6. Impact of Thawing Methods on Protein Oxidation in A. neglectus

Myofibrillar protein (MP) is the richest and most important protein in meat, mainly including tropomyosin, myosin, myofibrillar protein, actomyosin, and so on. MP constitute 55% to 60% of the total protein content in muscles [41]. The structure of MP is prone to change during the thawing process, affecting the properties of protein in food. Oxidative modifications in proteins can lead to alterations in their functional and structural properties, ultimately affecting the overall quality of the meat product [42]. The oxidative changes in MP were assessed to understand the impact of different thawing methods on protein oxidation in octopi. Understanding the specific oxidative changes in MP can provide insights into the impact of thawing methods on the quality and nutritional aspects of octopus meat.

**Figure 5:**
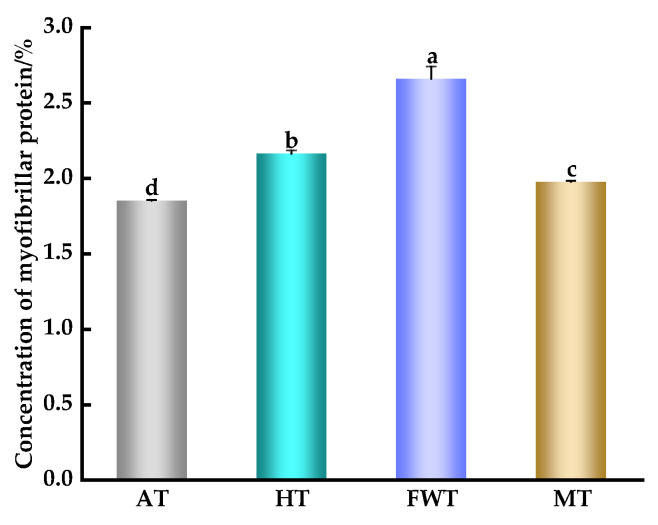
Effects of different thawing methods on the concentration of MP in *A. neglectus* (^a,b,c,d^ indicate that different thawing treatments have significant differences, *p* < 0.05).

As shown in Figure 5, MP extracted from octopus muscles exposed to different thawing methods revealed significant differences in MP concentration among the four groups (*p* < 0.05). The concentration of MP extracted from the FWT group was the highest, while the AT group had the lowest concentration. The difference between these two groups was significant (*p* < 0.05), with a variation of 0.81%. The observed differences in MP concentration could be attributed to the rapid thawing rate in the FWT group, minimizing MP damage and oxidation. In contrast, the AT group required a longer thawing period (7 h), leading to prolonged exposure to air, microbial proliferation, and protein oxidation, resulting in the lowest MP concentration.

**Figure 6:**
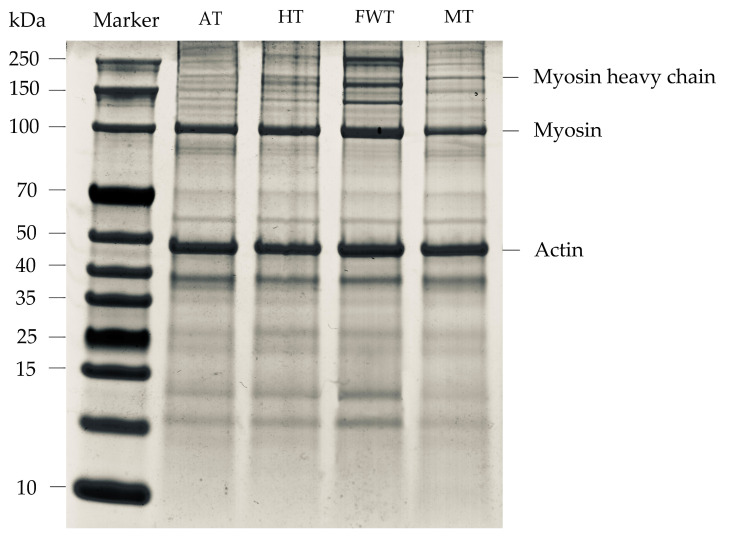
Changes in SDS–PAGE gel electrophoresis of MP in *A. neglectus* fillets in response to different thawing methods.

After extracting MP from octopus muscles, SDS–PAGE electrophoresis was performed to observe the relative molecular weights and the number of subunits in the protein molecules. The primary bands of MP include myosin heavy chain (MHC, 200 kDa), myosin (100 kDa), and actin (45 kDa) [43]. As depicted in Figure 6, the bands of MHC, myosin, and actin in the FWT group are broader compared to the bands in the AT, HT, and MT groups. This difference may be attributed to the restricted cross-linking of proteins under extreme conditions during AT, HT, and MT. Additionally, the oxidation of MHC, leading to the formation of disulfide bonds and carbonyl groups, could result in protein degradation and decreased solubility, leading to significant differences in band intensity in electrophoresis, particularly for MHC, which is more prone to protein degradation compared to other proteins, as observed in previous studies [44]. In a study on chicken MP electrophoresis, Zhan et al. [45] found that bands corresponding to myosin and actin disappeared more slowly. Furthermore, some bands appeared in the molecular weight range of 15–20 kDa, possibly due to cross-linking and aggregation of octopus MP during the thawing process, leading to denaturation and varying degrees of degradation [24].

The activity of Ca^2+^–ATPase can reflect the degree of denaturation and integrity of the myosin head, which contains the binding sites of Ca^2+^–ATPase activity, and thawing can lead to a varying degree of reduction in Ca^2+^–ATPase activity [46]. Therefore, Ca^2+^–ATPase activity can be used as an important indicator to evaluate protein oxidation. The sulfhydryl group (–SH) is one of the most active functional groups among the amino acid residues that make up proteins. They are exposed on the surface as the protein structure unfolds and can be oxidized into disulfide bonds. With the enhancement in oxidative conditions, sulfoxides and other oxidation products are generated, leading to a decrease in sulfhydryl group content. This indicates that the protein has undergone denaturation [47]. The sulfhydryl group plays a crucial role in stabilizing the spatial structure of MP. During the thawing process, the oxidation of sulfhydryl groups triggers the creation of disulfide bonds, resulting in the cross-linking, linking, and aggregation of protein molecules. Consequently, this brings about alterations in the spatial configuration of MP [48,49].

**Figure 7:**
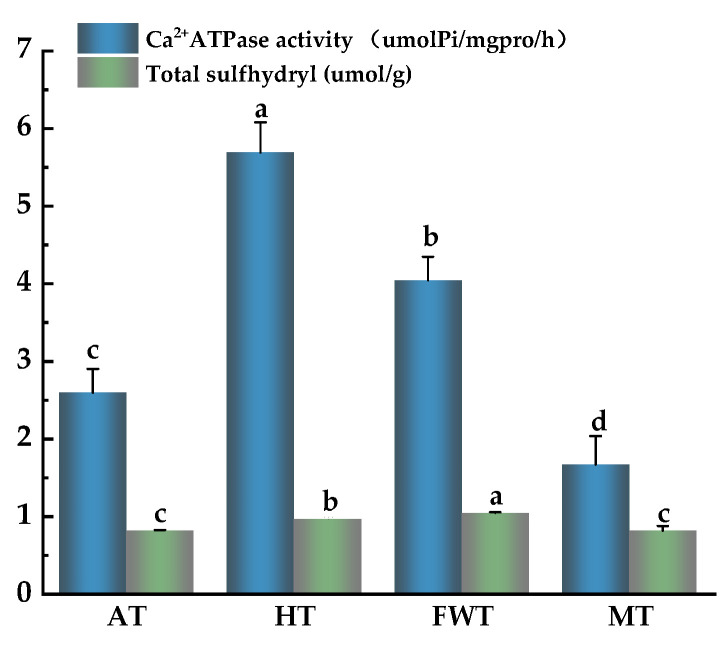
Effects of different thawing methods on the activity of Ca^2+^–ATPase and the total content of –SH in *A. neglectus* (^a,b,c,d^ indicate that different thawing treatments have significant differences, *p* < 0.05).

The impact of different thawing methods on octopus Ca^2+^–ATPase activity and total sulfhydryl content is illustrated in Figure 7. There were significant differences in Ca^2+^–ATPase activity and total sulfhydryl content among the four groups (*p* < 0.05). The FWT group had the highest total sulfhydryl content, which was 22.20%, 7.77%, and 22.04% higher than that in the AT, HT, and MT groups, respectively. Additionally, the Ca^2+^–ATPase activity was relatively high: 1.56 and 2.44 times higher than that in AT and MT groups, respectively. It was speculated that FWT can isolate the air, leading to lower protein oxidation levels, reducing losses in total sulfhydryl content and Ca^2+^–ATPase activity, and allowing better maintenance of the structural integrity of octopus muscle proteins. This trend aligned with the results obtained by Liu et al. [50].

In contrast, MT led to the lowest Ca^2^+–ATPase activity and sulfhydryl content, suggesting that the high temperature during MT treatment led to severe protein denaturation and degradation, substantially damaging the structure and integrity of protein, with a resulting decrease in Ca^2+^–ATPase activity and a sharp decline in sulfhydryl content [51]. However, protein oxidation was also found to be the least extensive in the FWT group, as evidenced by higher Ca^2+^–ATPase activity and greater sulfhydryl content, which are indicators of protein integrity.

## 4. Conclusions

This study demonstrates that different thawing methods significantly affected the texture, color difference, water retention, cooking loss, and protein content of octopus meat. Of all the evaluated methods, air thawing (AT) had the longest thawing time. Due to the long-term exposure to air and to relatively high temperatures, the probability of oxidative denaturation and degradation of protein was increased, and the TVB–N content was produced in significantly higher amounts than in the other three groups, with the lowest concentrations of MP and sulfhydryl content, the poorest springiness, and higher thawing and cooking loss rates. Overall, the quality indicators of octopus meat were lower after air-thawing. The thawing time of the HT group was significantly longer than that of the FWT and MT groups, leading to higher cooking loss rates and TVB–N content. However, immersion in hydrostatic water isolated some of the oxygen, alleviated the oxidation of protein to a certain extent, and maintained a higher level of Ca^2+^–ATPase activity, sulfhydryl content, and springiness. MT had the fastest thawing rate and the smallest TVB–N value, but exposure to uneven heating and excessive temperatures during this process caused the myofibrillar fibers of the octopus meat to contract and break, causing the meat to appear burnt, and resulting in an unfavorable color after thawing, in addition to the highest thawing and cooking loss rates. In contrast, FWT had the advantages of a short thawing time, minimum thawing loss rate and cooking loss rate, high content of non-flowing water, maximum water-holding capacity, and good water retention; the color of the octopus was excellent after the completion of this process (with high values of L^*^, *W* and b^*^). The concentration of MP in octopi was the highest after flowing-water thawing, with clearer subunit bands in SDS–PAGE electrophoresis, indicating lower degradation levels. It also maintained higher springiness, Ca^2+^–ATPase activity, and sulfhydryl content, suggesting that FWT had a demonstrable inhibitory effect on oxidation, alleviating protein oxidation degradation, and preserving higher quality characteristics. In conclusion, FWT emerged as the superior method for octopi, offering the best balance of speed and quality preservation. It effectively maintained water retention, color, pH, and protein quality, resulting in a product that is likely to be more appealing to consumers.

## Figures and Tables

**Table 1:** Experimental conditions of four thawing methods for frozen *A. neglectus*.

Thawing Methods	Operation Methods
Air thawing (AT)	A sealed bag containing frozen octopi was placed on a tray and thawed in ambient air at room temperature (24 ± 3) °C.
Hydrostatic thawing (HT)	A sealed bag containing frozen octopi was placed in a water bath to thaw at a constant temperature at (20 ± 1) °C with a sample–liquid ratio of 1:10.
Flowing-water thawing (FWT)	A sealed bag containing frozen octopi was thawed under a uniform tap water flow at a rate of 50 mL/s.
Microwave thawing (MT)	The frozen octopi were thawed in a microwave at a power range of 500–600 W.

**Table 2:** Effects of different thawing methods on the water holding capacity of *A. neglectus*.

Water Retention Index	AT	HT	FWT	MT
Time/min	400 ± 16.33 ^c^	115 ± 4.08 ^b^	39 ± 6.48 ^a^	20 ± 1.63 ^a^
Thawing loss rate/%	22.06 ± 0.76 ^b^	11.49 ± 1.68 ^a^	11.25 ± 0.78 ^a^	33.28 ± 2.30 ^c^
Cooking loss rate/%	42.65 ± 0.58 ^d^	36.34 ± 1.00 ^c^	26.50 ± 0.06 ^a^	32.75 ± 0.30 ^b^
Water-holding capacity/%	67.61 ± 2.23 ^c^	70.56 ± 5.67 ^bc^	76.94 ± 4.09 ^a^	74.25 ± 1.87 ^ab^

^a,b,c,d^ indicate that different thawing treatments have significant differences, *p* < 0.05.

**Table 3:** Effects of different thawing methods on the color and pH of *A. neglectus*.

Thawing Methods	AT	HT	FWT	MT
*L* ^*^	34.97 ± 0.83 ^b^	38.48 ± 5.33 ^ab^	40.30 ± 0.88 ^a^	31.43 ± 2.36 ^c^
*a* ^*^	4.99 ± 0.33 ^c^	7.01 ± 0.20 ^a^	6.11 ± 0.15 ^b^	6.82 ± 0.69 ^a^
*b* ^*^	9.02 ± 0.16 ^c^	10.33 ± 0.33 ^b^	11.01 ± 0.13 ^a^	8.07 ± 0.11 ^d^
*W*	34.16 ± 0.83 ^c^	37.22 ± 5.33 ^b^	38.99 ± 0.88 ^a^	30.62 ± 2.36 ^b^
pH	6.94 ± 0.02 ^a^	6.76 ± 0.02 ^b^	6.52 ± 0.01 ^d^	6.63 ± 0.02 ^c^

^a,b,c,d^ indicate that different thawing treatments have significant differences in each row, *p* < 0.05.

## Data Availability

The original contributions presented in the study are included in the article, further inquiries can be directed to the corresponding authors.
